# A Point of Care Ultrasound (POCUS) Artifact Mimicking an Aortic Dissection: A Case Series

**DOI:** 10.24908/pocusj.v10i01.18498

**Published:** 2025-04-15

**Authors:** Olivia Klee, Julia Buechler, Molly Fears, Caroline Gosser, Kahra Nix

**Affiliations:** 1 School of Medicine, University of Louisville, Louisville, KY, USA; 2 Department of Emergency Medicine, School of Medicine, University of Louisville, KY, USA

**Keywords:** abnormal aorta, incidental findings, aortic dissection, pseudo dissection, point of care ultrasound, POCUS

## Abstract

**Introduction:**

This case series describes a point of care ultrasound (POCUS) artifact involving the abdominal aorta of four standardized patients. The purpose of this case series is to highlight this artifact and maneuvers to discern pathology from normal.

**Methods:**

Permission was obtained for each case described in this series. POCUS images of the abdominal aorta in both sagittal and transverse were obtained in these four cases. The findings were reviewed and compared.

**Discussion:**

All four standardized patients were otherwise healthy, thin and female. The artifact was consistently a linear, hyperechoic structure within the lumen of the abdominal aorta in the sagittal plane.

**Conclusion:**

In each of these cases, the artifact disappeared on rotation of the probe from the sagittal plane to the transverse plane. Knowledge of this POCUS artifact and maneuvers to avoid it are important in both clinical and educational settings.

## Introduction

Point of care ultrasound (POCUS) is evaluated along with other knowledge, skills, attitudes and attributes in the Emergency Medicine Milestone as defined by the American College of Graduate Medical Education [1]. In a policy statement, the American College of Emergency Physicians (ACEP) acknowledged POCUS as a fundamental part of Emergency Medicine (EM) training [2]. In order to achieve these goals, EM residents have discrete and longitudinal POCUS training. Often, medical students are included in bedside teaching both in the operator role and as standardized patients. When acting as a standardized patient, verbal consent is obtained. The potential for incidental findings is acknowledged as a possibility with a clear plan for next steps [3]. In this case series, we describe an artifact that mimics a dissection involving the abdominal aorta that was found on a young, healthy, thin female medical student who was acting as a standardized patient. A radiology-performed ultrasound of her abdomen confirmed the abdominal aorta as normal. This same artifact was subsequently seen on three additional young, healthy, thin, female medical students.

## Methods

Each of the standardized patients in this case series provided written, informed consent for the POCUS images to be obtained after a full explanation was provided. The abdominal aorta of four standardized patients was evaluated using a Sonosite curvilinear (5-1 MHz) (Sonosite, Bothwell, WA, USA) or a Mindray curvilinear (C6-1s) transducer (Mindray, Mahwah, NJ, USA) by placing the probe in the sagittal and transverse planes along the abdomen aorta. Clips and stills of both the sagittal and transverse views of the abdominal aorta were recorded. This case series was completed after the University of Louisville Institutional Review Board determined it was exempt. Each of these standardized patients also consented to this case series.

## Case Descriptions and Results

### Case 1

**Figure 1.**
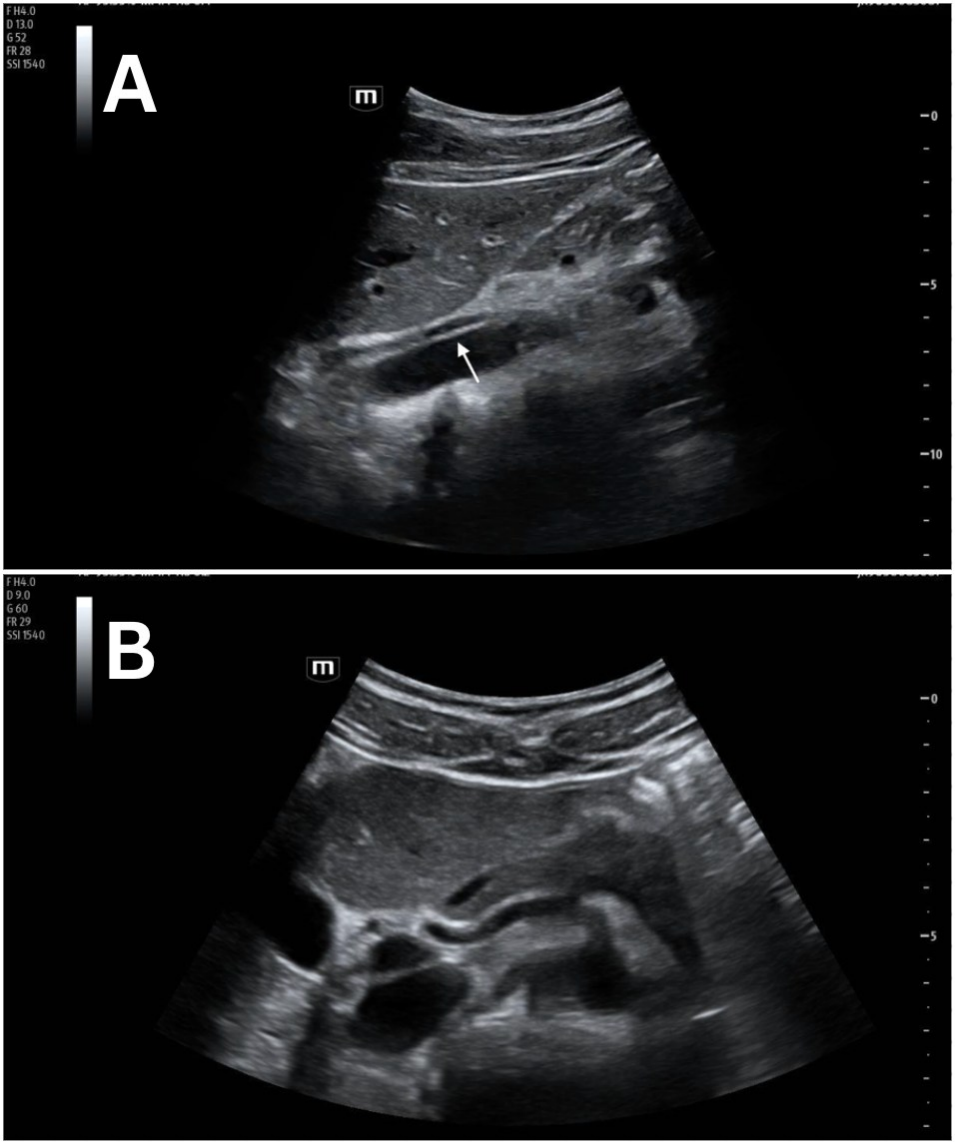
Grayscale POCUS images of a 22-year-old female standardized patient showing (A) sagittal view with a linear, hyperechoic structure within the lumen of the abdominal aorta and (B) transverse view with a normal, anechoic lumen of the abdominal aorta.

A POCUS examination was performed of the abdominal aorta of a 22-year-old medical student acting as a standardized patient. The study revealed a hyperechoic, linear structure within the lumen of the aorta in the sagittal plane that resolved on rotation of the probe into the transverse plane (Figure 1, Video 1).

### Case 2

**Figure 2.**
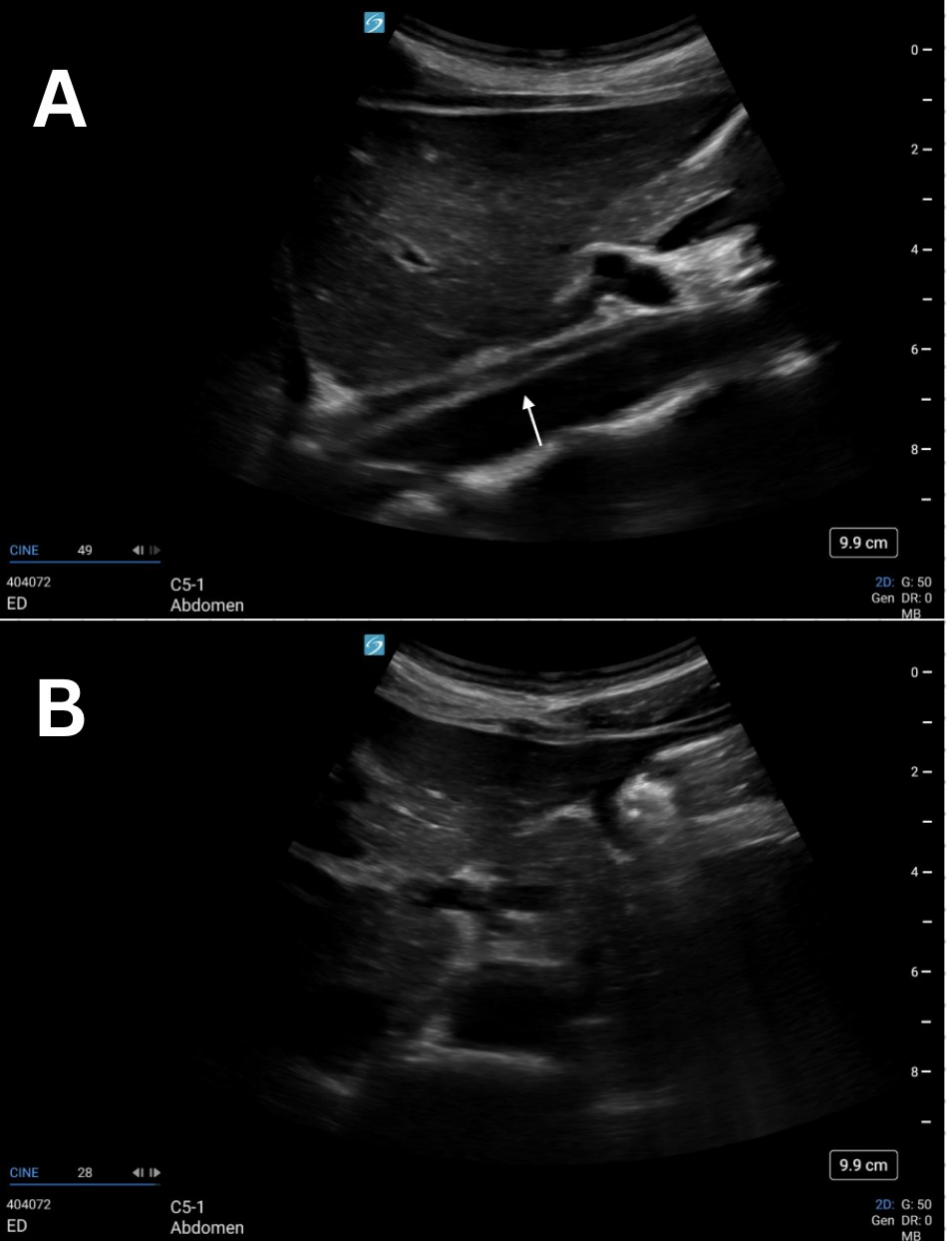
Grayscale POCUS images of a 25-year-old female standardized patient showing (A) sagittal view with a linear, hyperechoic structure within the lumen of the abdominal aorta and (B) transverse view with a normal, anechoic lumen of the abdominal aorta.

A POCUS examination was performed of the abdominal aorta of a 25-year-old medical student acting as a standardized patient. The study revealed a hyperechoic, linear structure within the lumen of the aorta in the sagittal plane that resolved on rotation of the probe into the transverse plane (Figure 2, Video 2).

### Case 3

**Figure 3.**
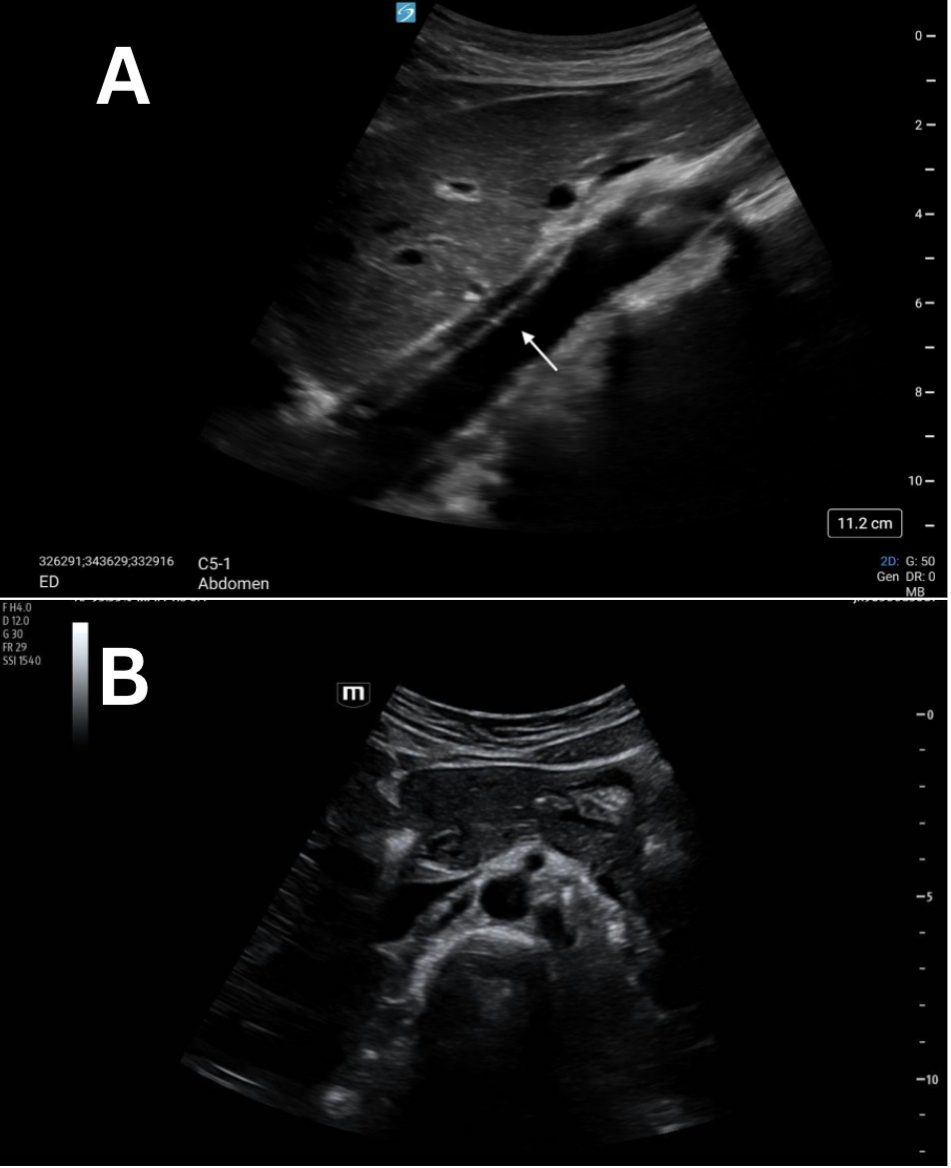
Grayscale POCUS images of a 27-year-old female standardized patient showing (A) sagittal view with a linear, hyperechoic structure within the lumen of the abdominal aorta and (B) transverse view with a normal, anechoic lumen of the abdominal aorta.

A POCUS examination was performed of the abdominal aorta of a 27-year-old medical student acting as a standardized patient. The study revealed a hyperechoic, linear structure within the lumen of the aorta in the sagittal plane that resolved on rotation of the probe into the transverse plane (Figure 3, Video 3).

### Case 4

**Figure 4.**
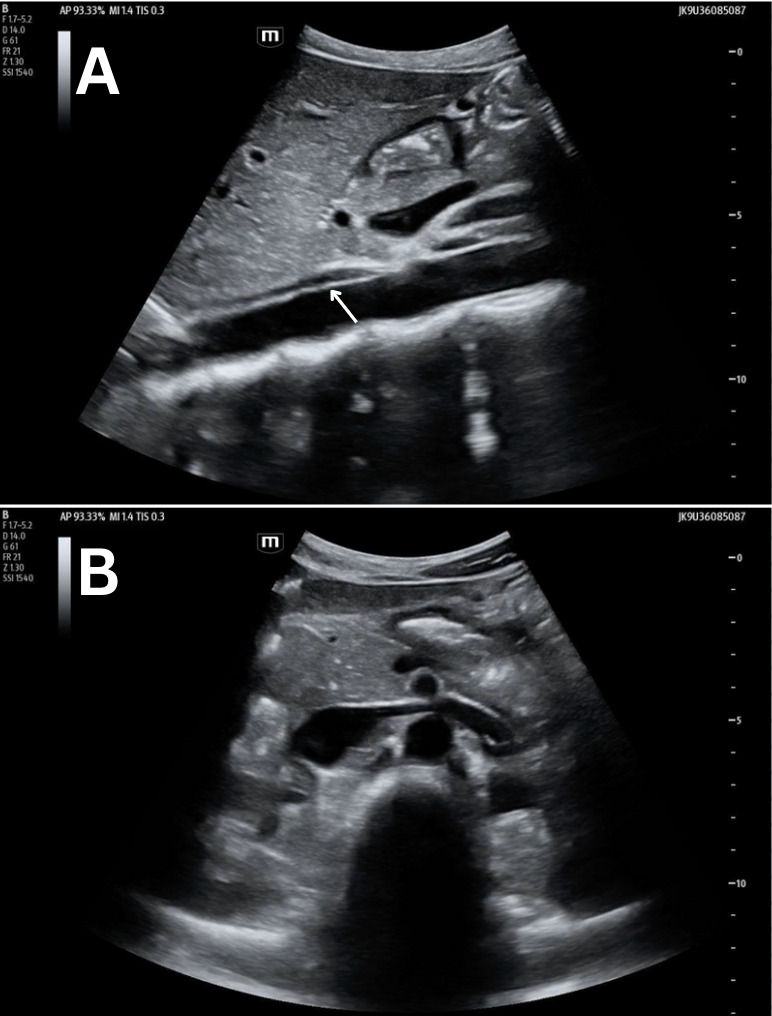
Grayscale POCUS images of a 25-year-old female standardized patient showing (A) sagittal view with a linear, hyperechoic structure within the lumen of the abdominal aorta and (B) transverse view with a normal, anechoic lumen of the abdominal aorta.

A POCUS examination was performed of the abdominal aorta of a 25-year-old medical student acting as a standardized patient. The study revealed a hyperechoic, linear structure within the lumen of the aorta in the sagittal plane that resolved on rotation of the probe into the transverse plane (Figure 4, Video 4).

## Discussion

POCUS artifacts are frequently encountered in both clinical and educational settings. Case 1 shows the initial artifact that was found involving the abdominal aorta of a young, healthy standardized patient. This standardized patient was sent for a radiology-performed ultrasound of her abdomen that confirmed her abdominal aorta as normal. This same artifact was subsequently seen on three additional young, healthy, thin, female medical students acting as standardized patients.

Previously described cases show discrete artifacts found within the lumen of the aorta mimicking both dissection and thrombus [4–7]. Our proposed etiology of this artifact is side-lobe artifact, which is known to happen within vessels. This can result in the appearance of an echogenic structure that did not actually originate from within the vessel. Consistent with other examples of side-lobe artifact, the linear, hyperechoic artifact in these four cases disappeared when rotating the probe from the sagittal to the transverse plane (Figures 1-4). These structures were further confirmed as artifacts since none undulated like an acute dissection of the abdominal aorta. Artifacts within vessels are also known to occur as a result of mirroring and reverberation. These resolve with rotation and/or translation of the probe.

## Conclusion

This case series highlights that when performing POCUS examinations, physicians and other providers must be aware of the potential for artifacts and have enough understanding of maneuvers to distinguish pathology from artifact. In abdominal aorta scanning in the sagittal plane (long axis of the vessel), side lobe artifact can mimic abdominal aorta dissection, but this is resolved when scanning in the transverse plane.








